# Identification of Biomarkers for Cervical Cancer Radiotherapy Resistance Based on RNA Sequencing Data

**DOI:** 10.3389/fcell.2021.724172

**Published:** 2021-08-03

**Authors:** Yue Feng, Zhao Wang, Nan Yang, Sijia Liu, Jiazhuo Yan, Jiayu Song, Shanshan Yang, Yunyan Zhang

**Affiliations:** Department of Gynecological Radiotherapy, Harbin Medical University Cancer Hospital, Harbin, China

**Keywords:** biomarkers, cervical cancer, radiotherapy resistance, bioinformatic, RNA sequencing data

## Abstract

Cervical cancer as a common gynecological malignancy threatens the health and lives of women. Resistance to radiotherapy is the primary cause of treatment failure and is mainly related to difference in the inherent vulnerability of tumors after radiotherapy. Here, we investigated signature genes associated with poor response to radiotherapy by analyzing an independent cervical cancer dataset from the Gene Expression Omnibus, including pre-irradiation and mid-irradiation information. A total of 316 differentially expressed genes were significantly identified. The correlations between these genes were investigated through the Pearson correlation analysis. Subsequently, random forest model was used in determining cancer-related genes, and all genes were ranked by random forest scoring. The top 30 candidate genes were selected for uncovering their biological functions. Functional enrichment analysis revealed that the biological functions chiefly enriched in tumor immune responses, such as cellular defense response, negative regulation of immune system process, T cell activation, neutrophil activation involved in immune response, regulation of antigen processing and presentation, and peptidyl-tyrosine autophosphorylation. Finally, the top 30 genes were screened and analyzed through literature verification. After validation, 10 genes (KLRK1, LCK, KIF20A, CD247, FASLG, CD163, ZAP70, CD8B, ZNF683, and F10) were to our objective. Overall, the present research confirmed that integrated bioinformatics methods can contribute to the understanding of the molecular mechanisms and potential therapeutic targets underlying radiotherapy resistance in cervical cancer.

## Introduction

Cervical cancer is the fourth most common malignancy and the fourth leading cause of cancer-related mortality in women globally (Barker et al., 2015; Doja et al., 2020; Sahu and Pattanayak, 2020). Among women between the ages of 20 and 39, cervical cancer is the second leading cause of cancer deaths (Siegel et al., 2020). To date, approximately 80% of patients with cervical cancer need radiotherapy in the process of treatment (Jemal et al., 2011). Radiotherapy resistance, also known as radiosensitivity, remains a significant hurdle to cervical cancer therapeutics. Since 1999, the cisplatin-based concurrent chemoradiotherapy (CCRT) is the standard of treatment for advanced cervical cancer (Rose et al., 1999). Compared with radiotherapy alone, CCRT increases patients’ local control rates and can improve prognosis. However, cisplatin increases the incidence of acute hematological toxicity, which may lead to the interruption of CCRT treatment and poor prognosis in patients with cervical cancer (Green et al., 2001; Dueñas-González et al., 2011; Bazan et al., 2013; Liu et al., 2020). The 5 years survival rate for advanced cervical cancer is still only 5–15% (Chopra et al., 2018). Therefore, exploring the mechanisms underlying radiosensitivity at the molecular level is the essential strategy for increasing long-term survival of cervical cancer.

Resistance to radiotherapy can be intrinsic or acquired, but its specific mechanism is not yet clear. As a promising predictive application, bioinformatics has led to breakthroughs in the field of medical research (Kanehisa and Bork, 2003; Wadlow and Ramaswamy, 2005; Oliver et al., 2015; Tang et al., 2018; Gauthier et al., 2019; Yang and Deng, 2020; Zhang, 2020; Zhang et al., 2020b). For decades, many bioinformatics studies have been performed to investigate radiotherapy resistance-related mechanisms and detect molecular biomarkers (Zhang et al., 2020c,d). However, in radiotherapy, a tumor may gradually adapt to changes in physical and chemical environments and acquire radiotherapy resistance (Baskar et al., 2012; Stapleton et al., 2017). Reliable studies that focus on changes in gene expression during radiotherapy treatment based on bioinformatics are few. Hence, key genes for revealing molecular mechanisms to overcome cervical cancer radiotherapy resistance according to genomic changes should be identified.

In the current study, we acquired a cervical cancer-associated gene expression dataset, comparing cervix cancer tissue between pre-irradiation and mid-irradiation status to investigate the phenomenon of changes in gene expression during radiotherapy. Second, we performed gene expression profile analysis and identification of differentially expressed genes (DEGs). Correlations between these genes were discovered through the Pearson correlation analysis. Subsequently, random forest was used in optimizing risk genes. According to the random forest scoring results, the top 30 genes were selected as candidate genes for verification analysis. Finally, a total of 10 signature genes were verified through comprehensive literature searching as biomarkers for radiotherapy resistance in cervical cancer.

## Materials and Methods

**FIGURE 1:**
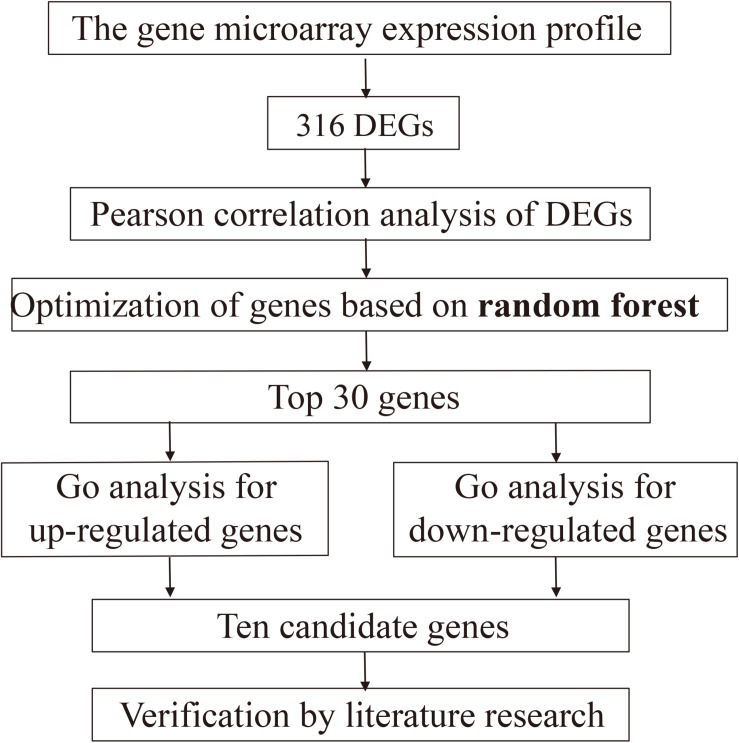
The overall workflow.

The whole designation was performed according to the flow chart (Figure 1).

### Microarray Data Collection and Preprocessing

The gene expression profile was downloaded from the Gene Expression Omnibus (GEO) database^1^ which includes a variety of gene expression status of public genomics data (Dueñas-González et al., 2011). We retrieved the keyword “cervical cancer” in this functional database to obtain relevant information about radiotherapy resistance. The inclusion criteria were as follows: (a) the sample was treated with CCRT and without neoadjuvant chemotherapy; (b) number of samples were not less than 50; and (c) sample data were reliable.

The raw GEO dataset of GSE3578 was downloaded as the validation set. It had a total of 156 samples (78 pre-irradiation and 78 mid-irradiation samples). The dataset was based on the platform GPL2895, GE Healthcare/Amer sham Biosciences Code Link Human Whole Genome Bio array. Next, we preprocessed the data and converted each probe ID into a gene symbol. Probes without corresponding or mapping to multiple gene symbols were rejected to prevent ambiguity (Zhang et al., 2018).

### Gene Expression Profile Analysis and Identification of Differentially Expressed Genes

The limma package was used in processing the downloaded expression profile and converting and filtering unqualified data to obtain DEGs (Ritchie et al., 2015; Liu et al., 2019b). The original data were chip data, and thus the limma package was used for our analysis. DEGs between the pre-irradiation and mid-irradiation samples were identified, and a *P* value of <0.05 and | log2fold change (FC)| of >0.5 were used as the cutoff thresholds.

### Pearson Correlation Analysis

Pearson correlation analysis is a statistical method with powerful functions for accurately analyzing gene or protein co-expression correlations (Zhang et al., 2020a; Tang et al., 2021). In this study, it was used in assessing the relationships among the 316 DEGs obtained. According to the results of the analysis, genes with Pearson correlation coefficient threshold absolute values of >0.8 were selected as candidate genes.

### Random Forest

Random forest is a generally acknowledged ensemble classifier for machine learning and can exploit large data repositories for the analysis of risk predictors and their intimate interactions and advancement risk prediction capability (Yang et al., 2021). It was used in evaluating cervical cancer candidate genes as the main objects of our research (Nunn et al., 2012; Wang et al., 2018; Deng et al., 2019b; Ru et al., 2019; Lv et al., 2020; Yang et al., 2020). In the random forest setting, we divided the expression profile data of 103 genes according to the properties of the samples and considered the samples before radiotherapy as “good” and the samples during radiotherapy “poor,” respectively. Then, 156 samples from GSE3578 were randomly divided into three groups. One group (52 samples) was used as the training set, whereas the other two groups (104 samples) were as the test sets. All samples were used for random forest scoring (Rigatti, 2017; Wang and Zhou, 2017).

### Enrichment Analysis

Gene ontology (GO) enrichment was performed for the annotation of the three types of functions of genes, namely, biological process (BP), cytological component, and molecular function (Peng et al., 2017; Deng et al., 2019a). The gene list containing the top 30 scores in the random forest was regarded as an input file and utilized for GO function enrichment in the R package. On the basis of the reconstructed enrichment analysis, the results of pathways on cervical cancer radiosensitivity were further found.

### Screening and Analysis of the Top 30 Genes

Medical literature retrieval is the basic and authoritative verification method in the verification process. After obtaining 30 candidate genes, we extensively reviewed the literature to determine whether the selected genes are indeed signature genes for cervical cancer radiotherapy resistance. We retrieved the name of the genes with the keywords “cervical cancer,” “radiotherapy resistance,” “irradiation,” and “radiosensitivity” in the databases to obtain relevant information about the top 30 genes. Ultimately, we obtained the signature genes for cervical cancer radiotherapy resistance through comprehensive analysis.

## Results

### Identification of Differentially Expressed Genes

First, to investigate the radiosensitivity in cervical cancer, we processed and converted the raw gene microarray expression dataset GSE3578 associated with the cancer. We extracted 17,097 genes from the obtained microarray dataset. Significance analysis with the limma package indicated that 316 DEGs were present in the samples, of which 237 were obviously down-regulated and 79 were up-regulated.

### Pearson Correlation Analysis of DEGs

**FIGURE 2:**
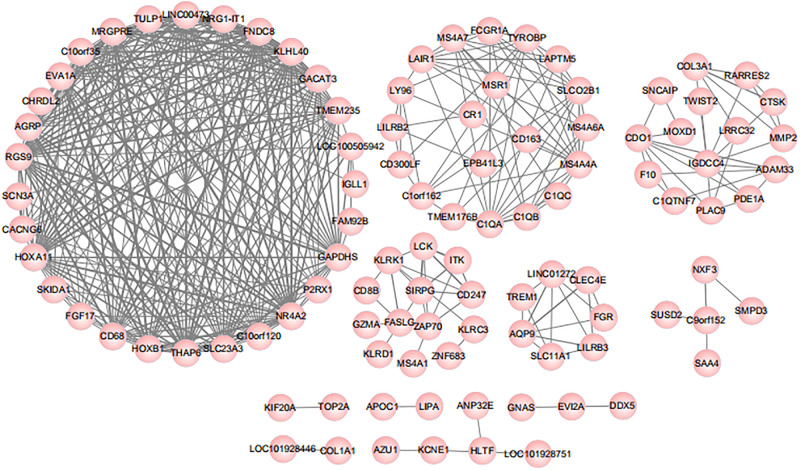
Results of Pearson correlation analysis. According to the Pearson correlation coefficient, we obtained the correlations between genes, that is, the greater the connectivity, the greater the node size of the gene displayed.

Pearson correlation analysis was subsequently performed between 316 differential genes for the assessment of the association (Cheng et al., 2019). Genes with a correlation coefficient threshold greater than 0.8 in absolute value were selected on the basis of the results. The analysis results were provided in Figure 2, and 103 genes met the threshold setting requirements. All the small nodes were the differential genes we calculated and each node represented a gene with a correlation coefficient greater than 0.8. According to the Pearson correlation coefficient, we obtained the correlations between genes, that is, the greater the connectivity, the greater the node size of the gene displayed.

### Optimization of Candidate Genes With Random Forest

Random forest classification is a universally accepted machine learning procedure for regression tree classification and regression and model prediction (Cheng et al., 2018; Sapir-Pichhadze and Kaplan, 2020; Ubels et al., 2020; Zhao et al., 2020). It was first proposed by Breiman in 2001. It takes advantage of binary splits on predictor variables for value outcome forecasts (Steffens et al., 2006). To achieve cervical cancer-related candidate genes, we built a random forest model. We divided the 156 samples into three parts, of which one was the training set (52 samples) and the other was the test set (104 samples). In the test set, the “good group” contained 51 samples, meanwhile, the “poor group” contained 56 samples. Regarding the differential genes as features, the test set was classified by random forest method. When the number of “ntrees” was 1,000, we obtained the most ideal training result with a classification error rate of 9.62%, of which five cases were misclassified in the “good group” and five cases were misclassified in the “poor group.” As the results were obtained through the classification of the test set, we regarded this classified procedure noble ideal.

Furthermore, we comprehensively utilized random forest scoring on all samples to further verify the accuracy of our data. The results revealed that the minimum error rate of the classification results was only 1.92% and 78 samples were in the “good group” and one was misclassified. The “poor group” contained 78 samples, including two misclassified samples. After several times of analysis, the error rate of random forest classification results was less than 5%, indicating that the differential genes as features can classify samples well. Therefore, sample classification with the genes had a low error rate, indicating that our classification results were of great significance and had the potential as molecular markers of cervical cancer radiotherapy resistance.

**FIGURE 3:**
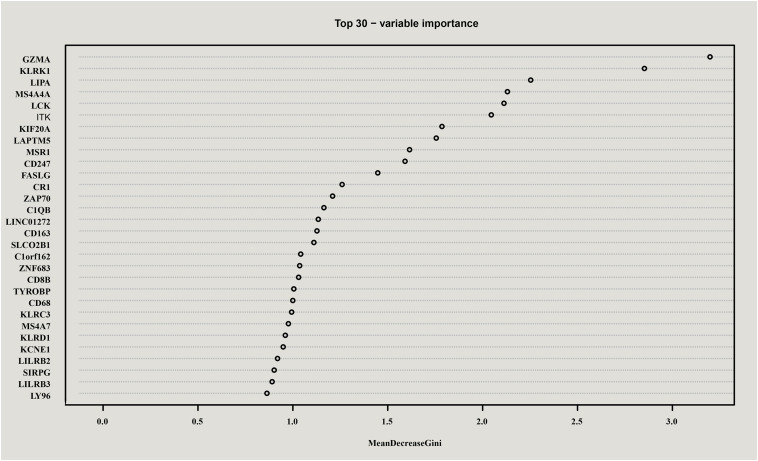
Top 30 genes in random forest scoring results. All genes were sorted in descending order according to Mean decrease gini (MDG), and the top 30 candidate genes for radiotherapy resistance in cervical cancer were selected.

Mean decrease gini (MDG), one of importance measures in random forest scoring, was employed to rank variables and for variable selection. In this study, MDG marked the degree of contributions of genes that could distinguish the characteristics of samples before and during radiotherapy. As a result, all genes were sorted in descending order according to MDG, and the top 30 candidate genes for radiotherapy resistance in cervical cancer were selected and shown in Figure 3.

### Enrichment Functional Analysis of Candidate Genes

**FIGURE 4:**
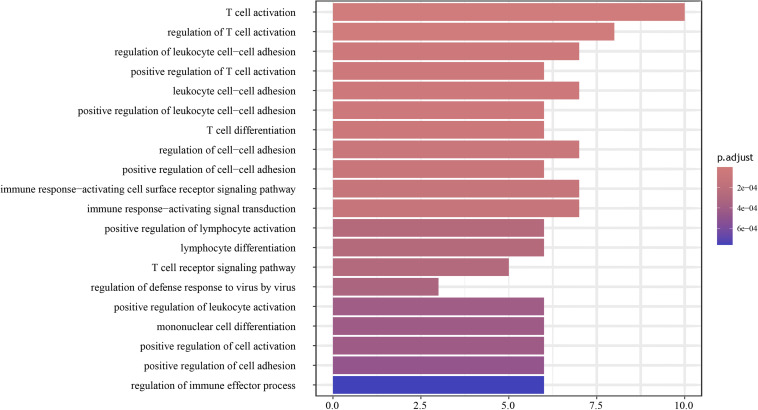
Enrichment analysis results of biological process (BP) was constructed by R package. The biological functions chiefly enriched in tumor immune responses.

To further uncover the molecular biology functions regulated by the genes, the top-ranked 30 genes according to the random forest scoring results were subsequently employed for the functional analysis of BP. As shown in Figure 4, the results indicated that biological functions were chiefly enriched in the corresponding tumor immune process pathways with the high fold overexpression, including cellular defense response, negative regulation of immune system process, T cell activation, neutrophil activation involved in immune response, regulation of antigen processing and presentation, and peptidyl-tyrosine autophosphorylation. These immune biological functions are significantly correlated with the examination of radiotherapy resistance. Researches on the radiotherapy resistance of cervical cancer from the perspective of the tumor immune microenvironment is extensive (Barker et al., 2015), showing that tumor radiotherapy has a certain impact on tumor microenvironment (TME), and changes in the TME, such as the generation of tumor-associated macrophages (TAMs) and cancer-associated fibroblasts can in turn affect the sensitivity of radiotherapy (Chu et al., 2014; Zhang et al., 2017; Choo et al., 2018). This shows that our enrichment results are very valuable. Given that the satisfying pathways were enriched, we used the results for the follow-up verification of the signature genes.

### Validation of Candidate Genes in Literature Retrieval

Considering the specific functions of the candidate genes in the sensitivity of cervical cancer radiotherapy, we comprehensively conducted a systematic medical literature search based on the top 30 genes. After verification in the literature, the candidate genes ranked 1, 5, 7, 9, 11, 13, 14, 18, 24, and 29 were confirmed with relevant experiments with cancers, demonstrating that these genes were our signature genes for cervical cancer radiotherapy sensitivity. In details, Killer cell lectin like receptor K1 (KLRK1), lymphocyte protein tyrosine kinase (LCK), kinesin family member 20A (KIF20A), CD247 molecule (CD247), Fas ligand gene (FASLG), CD163 molecule (CD163), zeta-chain associated protein 70 (ZAP70), CD8b molecule (CD8B), zinc finger protein 683 (ZNF683), and coagulation factor X (F10) were the top 10 genes. The scores of the 11th and 13th genes were closely associated to cervical cancer radiotherapy resistance, the 7th, 24th, and 29th genes were associated with cervical cancer, the first gene was correlated with cervical intraepithelial neoplasia, the 5th, 14th, and 18th genes were related to radiosensitivity for other cancers, and the 9th gene was associated to tumor immune microenvironment. In conclusion, this suggests that signature genes are expected to be biomarkers for cervical cancer radiotherapy resistance based on RNA sequencing data.

## Discussion

Although radiotherapy for cervical cancer has been widely used, the emergence of radioresistance has deeply affected its treatment effect (Kaidar-Person et al., 2012; Han et al., 2021). Consequently, overcoming the radiotherapy resistance of cervical cancer and finding related accurate biomarkers are essential and urgently needed. Different gene expression characteristics may lead to different types of tumors with varying degrees of sensitivity to radiotherapy (Jiang et al., 2017; Prevo et al., 2017; Zhou et al., 2017). With the development of bioinformatics, gene expression microarrays have been widely used in studying cancer-related genes and considered promising tools for molecular prediction (Cheng et al., 2017; Liu et al., 2019a; Xing et al., 2020).

In the present study, the data from GSE3578 were used in identifying 316 DEGs, which we regarded as the origins. The relationships among these genes were considered, and Pearson correlation analysis was employed. A total of 103 genes with correlation coefficient thresholds >0.8 in absolute value were used for subsequent gene optimization. Identifying cancer-related candidate genes through the random forest method is important. The expression profile data of 103 genes were classified according to the properties of the samples. Then, the 156 samples in GSE3578 were randomly divided into a training set and two test sets, and all the samples were used for random forest scoring. The error rate of sample classification based on these genes was sufficiently small, which can help us to distinguish between normal and disease samples.

Gene ontology term enrichment analysis was conducted using the top 30 genes for the recognition of the roles of DEGs in radiotherapy initiation and progression in cervical cancer. The BP enrichment results were closely related to radiotherapy resistance in cervical carcinoma, suggesting that cervical cancer radiotherapy and immune system are mutually influential. That is, these results showed the specific molecular mechanisms of radiosensitivity in cervical cancer to a certain degree. According to the enriched GO terms, these genes were selected for subsequent verification and analysis. After thoroughly verifying the above genes in the literature, we observed that 10 genes were significant. These genes were selected as our signature genes for cervical cancer radiotherapy sensitivity.

Many studies have been carried out on the 10 candidate genes, and the ranking of the 11th and 13th genes were the most meaningful for our purpose. First, the genes related to cervical cancer or radiotherapy sensitivity were explored. FASLG, a type II membrane protein in the tumor necrosis factor superfamily of death receptors, can combine with and trimerize the death receptor Fas on cell surfaces and thereby activate authoritative extrinsic pathways for apoptosis (Nagata, 1997; Fulda and Debatin, 2006). Cervical cancer is sensitive to radiotherapy because this gene has different levels of expression. Di et al. stated that the proportion of the pro-apoptotic protein FASLG is up-regulated by the radiotherapy sensitizer diallyl disulfide, which induce the activation of the apoptotic pathway in HeLa cells; they also showed that FASLG plays a direct role in the study of human cervical cancer cell apoptosis induced by radiosensitizers (Fulda and Debatin, 2006). In addition, radiotherapy plays an important role in the adjuvant treatment of glioma cancer; Huang et al. (2019) showed that the radiosensitivity of human glioma cells increases through the FASLG expression-associated signaling pathway. FASLG may be a candidate signature for the mechanism of radiotherapy response in cervical cancer.

Changes in the TME are closely related to the biological effects of radiation and are important factors that affect the sensitivity of tumor cells to radiotherapy (Barker et al., 2015). CD163, one of the immune cell markers for macrophages in the TME, is often used in determining M1 and M2 macrophages (de Vos van Steenwijk et al., 2013). The biological behavior of FASLG is closely correlated with the radiosensitivity of cervical cancer. Lippens et al. (2020) found that patients with reducing CD163 scores showed more pathological complete response, besides, patients with high levels of CD163 cells before treatment that were decreased after therapy had better prognosis. Another study focused on impacts on TME subjected to radiotherapy against head and neck squamous cell carcinoma and pointed out the up-regulation of M2 macrophage-related genes (CD163 and CD206) in irradiated tumors (Moreira et al., 2021). M2 phenotype TAMs are associated with decrease in disease-free survival in advanced breast cancer, and the M2-shifted subgroup is unsuitable for partial breast irradiation (Schnellhardt et al., 2020). Radiation therapy, in addition to killing cancer cells, affects the TME, particularly contributing to tumor blood vessel damage and immunosuppressive pathway activation (Jarosz-Biej et al., 2019). The accumulation of cells can lead to the suppression of radioactive response specifically in TAMs with the M2 phenotype, which facilitates the survival of cancer cells (Fridman et al., 2017). Previous reports and our results have pointed out that CD163 may have a key role in exploring the effectiveness of radiotherapy in cervical cancer radiotherapy.

Four of the 10 candidate genes have been studied in cervical cancer, but their correlations with cervical cancer radiotherapy is unclear (Verhey and Hammond, 2009). KIF20A has a microtubule-dependent force, participating in different cellular processes, such as the formation of the biological behavior of spindle structures and chromosome composition. Overexpressed KIF20A increases the risk of cervical cancer. Through bioinformatics methods and meta-analysis, KIF20A expression was found to significantly vary among 363 normal tissues and cervical cancer tissues and thus considered correlated with cervical cancer development (Wu et al., 2018). He et al. (2020) detected the gene and protein expression levels of KIF20A and found that the up-regulation of KIF20A expression is positively associated with the proliferation and invasion of cervical cancer cells. KIF20A affects the proliferation, migration, and invasion of cervical cancer by participating in a new type of circ_0005576/miR-153-3p/KIF20A axis (Ma et al., 2019), and KIF20A expression is involved in HPV infection, clinical stage, tumor recurrence, pelvic lymph node metastasis, and poor outcomes in early-stage cervical squamous cell cancer (Zhang et al., 2016). Thus, aberrant KIF20A expression is a potential independent prognostic factor for cervical cancer and is correlated with other cancers; for instance, radiation resistance may be enhanced partly by inducing the expression of KIF20A (Xiu et al., 2018). Irradiation-induced IF20A overexpression is associated with liver fibrosis, and KIF20A is associated with advanced pancreatic cancer (Taniuchi et al., 2005). Therefore, KIF20A-targeted treatment may be of great significance and may serve as a potential method for cervical cancer or its radiotherapy.

ZNF683, as a transcription factor, disrupts transcriptional processes in tissue-resident memory T and natural killer T cells, supplying immune protection when the body is re-infected (Van Gisbergen et al., 2012; Mackay et al., 2016). Liu et al. (2018) conducted analysis to clarify the potential mechanism of biological behavior factors that cause cervical cancer. Their results elucidated that the ZNF683 mRNAs are linked to the GO term “adaptive immune response.” This result is consistent with ours. In our study, based on the gene list of the top 30 scores in the random forest model, GO function enrichment was constructed using the R package for the prediction of pathways on cervical cancer radiosensitivity and pathways related to immune response. ZNF683 probably mediated the regulation of immune response in malignant cervical cancer. F10 gene may lead to the malignant transformation of the hydatidiform mole and occurrence of gynecological malignancies (Zhang and Pang, 2017). In patients with F10 overexpression, poorly differentiated cervical cancer tissues are higher in proportion compared with well-differentiated cancer tissues, and the negative correlation may be related to the occurrence and development of cervical cancer. KLRK1 encodes NKG2D, serving as an activating receptor expressed by NK cells and T cell subsets (Raulet, 2003). KLRK1 was thought to promote cervical cancer and CIN lesion susceptibility in 195 patients from southern Brazil.

Although not related to cervical cancer, most of the other parts of the genes we obtained through bioinformatics analysis were found to be related to radiosensitivity for other cancers. LCK was verified as a member of the Src family of non-receptor tyrosine kinases (Veillette et al., 1991). In Chinese hamster V79 cells, the expression of LCK gene is a key response to ionizing radiation-induced apoptosis and increases with radiation dose and incubation period. The Src-like tyrosine kinase p56/Lck is involved in the regulation of apoptosis caused by ionizing radiation. Similarly, in radioresistant B-lymphoma cells, p56/Lck promotes radiation-associated cell apoptosis (Waddick et al., 1993; Belka et al., 1999). The B-cell receptor-associated Src-like kinase Btk exhibits this function in B cells (Uckun et al., 1996).

ZAP70 is involved in T cell receptor signal transduction. The ability of T lymphocytes to convert signals to the nucleus is essential to the initiation and preservation of immune response, and the stimulation of T-cell receptors requires ligands to stimulate events in cells organized by ZAP70 and other proteins (Mozaffari et al., 2007). According to our results, ZAP70 plays an important role in radiotherapy-related immune response pathway. Similarly, a longitudinal study focused on immunologic system effects of adjuvant chemotherapy and radiotherapy in breast cancer showed that regulatory T cells and ZAP70 remain in their low grade forms after adjuvant chemoradiotherapy. In radiotherapy for colorectal cancer, through RNA extraction and following bioinformatic analysis, overexpressed ZAP70 was found to influence prognostic factors, such as tumor size and lymph node metastasis and differentiation; this finding showed that ZAP70 gene may act as a sensitivity biomarker for radiotherapy in colorectal cancer (Huang et al., 2011). Most of our information about PD-1 signaling was obtained through the exploration of activated T cells. In addition, the inhibition of ZAP70 impedes the activation of transcription factors in PD-1 signaling transduction during the stimulation of T cells (Wu et al., 2019).

As for CD247 and CD8B, related reports have been published. Mori et al. (2021) performed univariate experiments and analysis and found that reduction in PD-L1 mRNA/CD8B mRNA in tumor tissues may be a specific prognostic factor for salvage surgery for local recurrence after definitive concurrent radiotherapy and chemotherapy. CD247, also called CD3z-chain or TCR-Z, is a 16 kDa molecule participating in the TCR complex (Germain and Stefanová, 1999). Abnormal CD247 causes the irregular stimulation of T cells upon appointment with the TCR. CD247 down-regulation in TILs and peripheral blood lymphocytes is related to several types of cancers, including gastric carcinoma, head and neck cancer, and ovarian cancer, suggesting that CD247 is a therapeutic target for these cancer types (Ishigami et al., 2002; Ye et al., 2019). However, the relationships of the four genes with cervical cancer are rarely investigated despite that they may play vital role in cervical cancer. Additional tests are needed to verify the mechanism of these genes in the radiosensitivity of cervical cancer.

We employed Pearson correlation analysis to assess the meaningful relationships among the DEGs. Random forest was used in revealing the error rate of classifying samples. The results are satisfying and suitable for distinguishing cervical cancer-related genes from the samples. The results of the top 30 gene enrichment pathways have been verified by medical literature search to confirm their correlation with the sensitivity of cervical cancer radiotherapy, indicating that our results are precise. Notably, the medical literature search further confirmed that the expression levels of the 10 risk genes are associated with cervical cancer radiotherapy response or different types of tumors to a certain degree. Hence, the results suggested that our analytical application is of great value in identifying radiotherapy resistant molecular biomarkers for cervical cancer.

However, our current study has limitations and drawbacks. In this research, we verified the top 30 genes through literature searching because this method is highly comprehensive and can be easily conducted. In subsequent studies, we will perform accurate experimental techniques in pre-irradiation and mid-irradiation cervical cancer cell lines to validate the mRNA and protein expression levels of the 10 signature genes in cervical cancer. According to the results of BP analyses, we concluded that our candidate genes were mainly abundant in the immune responses of tumors, which is a new perspective for our further study of the mechanisms. We will preserve on valuable researches of cervical cancer radiotherapy resistance to explore the potential therapeutic targets for it.

## Data Availability Statement

Publicly available datasets were analyzed in this study. This data can be found here: https://www.ncbi.nlm.nih.gov/geo/query/acc.cgi?acc=GSE3578.

## Author Contributions

YF and YZ project design. NY and ZW project supervision. YF, SL, JY, and JS data generation and analysis. YF and YZ revisions of the manuscript. All authors reviewed the manuscript.

## Conflict of Interest

The authors declare that the research was conducted in the absence of any commercial or financial relationships that could be construed as a potential conflict of interest.

## Publisher’s Note

All claims expressed in this article are solely those of the authors and do not necessarily represent those of their affiliated organizations, or those of the publisher, the editors and the reviewers. Any product that may be evaluated in this article, or claim that may be made by its manufacturer, is not guaranteed or endorsed by the publisher.

## Footnotes

1
https://www.ncbi.nlm.nih.gov/gds/
